# Template-free synthesis of polystyrene monoliths for the removal of oil-in-water emulsion

**DOI:** 10.1038/s41598-017-06572-7

**Published:** 2017-07-26

**Authors:** Guowei Wang, Bin Yu, Shiguo Chen, Hiroshi Uyama

**Affiliations:** 1 Nanshan District Key Lab for Biopolymers and Safety Evaluation, Shenzhen Key Laboratory of Polymer Science and Technology, Guangdong Research Center for Interfacial Engineering of Functional Materials, College of Materials Science and Engineering, Shenzhen University, Shenzhen, 518060 P.R. China; 2 Key Laboratory of Optoelectronic Devices and Systems of Ministry of Education and Guangdong Province, College of Optoelectronic Engineering, Shenzhen University, Shenzhen, 518060 P.R. China; 3 Department of Applied Chemistry, Graduate School of Engineering, Osaka University, Suita, 565-0871 Japan

## Abstract

Oil-in-water emulsions are harmful to both humankind and environment. Frequent oil spill disasters make it urgent to develop low cost and high-efficiency materials for the treatment of oil-in-water emulsions. In this study, we report the facile template-free synthesis of macroporous polystyrene (PS) monolith from PS solution using a thermally-induced phase separation (TIPS) technique. The fabricated monolith showed high hydrophobicity, superoleophilicity, and macroporous structure. Moreover, the monolith exhibited high removal efficiency toward different oil-in-water emulsions. The monolith can be fabricated from cheap and commonly-used plastic. Thus, we anticipate that this research will contribute to both the recycling of PS and the treatment of oil spill accidents.

## Introduction

Oil spill accidents can cause serious environmental pollution to ocean or coastal water. An example is the Gulf of Mexico oil spill accident occurred in 2010. In this accident, plenty of crude oil has been released to the environment. Without any treatment, the spilled oil can last for many years, making incessant damage to the environment^1^. Compared with the oil that forms a separated layer from water, oil-in-water emulsion is much more difficult to be removed. Hence, there are increasing requirements to develop an economic, facile, and effective method to remove oil-in-water emulsion.

Some engineering techniques have been developed for the oil spill clean-up, such as, *in situ* burning^2–4^, dredging^5^, bioremediation^6–8^, skimming^9^, dispersants^10, 11^, and solidifying. However, these methods are less effective for the removal of oil-in-water emulsion, and face the problem of high side effect toward the environment. This inspired material scientists to develop new absorption materials that have the potential for the treatment of oil spill accident. The new material should be hydrophobic^12–14^ to avoid the invading of water. In another hand, the new material should be oleophilic to adsorb the oils. Some new materials have been developed for example, superhydrophobic melamine sponge^15^, marshmallow-like macroporous gel^16, 17^, flexible polypropylene sponge^18^, carbon nanofiber aerogel^19, 20^, carbon nanotube sponge^21, 22^, spongy graphene^23^, and antifouling membrane^24^.

Polystyrene (PS) is one of the most widely used plastics, with an industrial production of several billion kilograms per year. PS is a major component of plastic debris in the ocean, and a main reason for white pollution. Because of its hydrophobic and oleophilic properties, PS shows high potential to be used as oil absorbent. A monolithic PS material with high surface area is highly demanded in the absorption application. To our best knowledge, porous PS monolith has never been synthesized from its polymers. The reported PS monoliths were synthesized from monomers (styrene) using template^25^. Apart from its high cost, the current method cannot contribute to the recycling of used PS.

To solve the above problems, herein we present for the first time the fabrication of a PS monolith using the thermally-induced phase separation (TIPS) technique^26–31^. The monolith was applied for the removal of oil-in-water emulsion in the low concentration range. We anticipated this new fabrication method will contribute to both the recycling of PS and the treatment of oil spill accidents.

## Results and Discussion

### Fabrication of PS monolith

**Figure 1:**
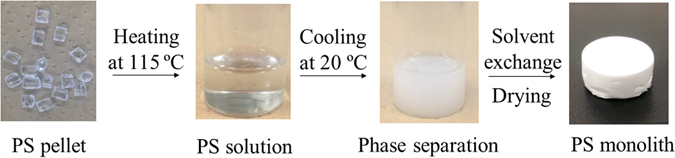
Fabrication procedure of a PS monolith.

A mixture of decalin and 1-butanol was used as the solvent for the fabrication of PS monolith (Fig. 1). PS pellet was dissolved in the solvent at 115 °C to form a homogenous solution. Subsequently, the solution was cooled at 20 °C. In the cooling process, the solution transferred from transparent to white color, which indicated the formation of the monolith. The PS monolith was immersed into 2-propanol to remove the embedded molecule and dried under vacuum. For the industrial application, cheap raw materials and a simple fabrication process are highly demanded. In this research, the polystyrene is low cost, and the fabrication process is simple and convenient, indicating that the method is very suitable for industrial production.

Both the solvent ratio and polymer concentration have great effects on the formation of a PS monolith^32^. A porous monolith can be formed when the decalin ratio range from 40 to 70%. In this paper, all the samples were prepared in the mixed solvent with fifty percent of decalin. The PS monolith can be formed if the polymer concentration is from 100 to 170 mg/mL. In case of a low concentration, the amount of polymer chains in a defined space are not enough to form a firmly connected structure. If the polymer concentration is too high, the high viscosity of the polymer solution makes it hard to form a porous structure.

**Figure 2:**
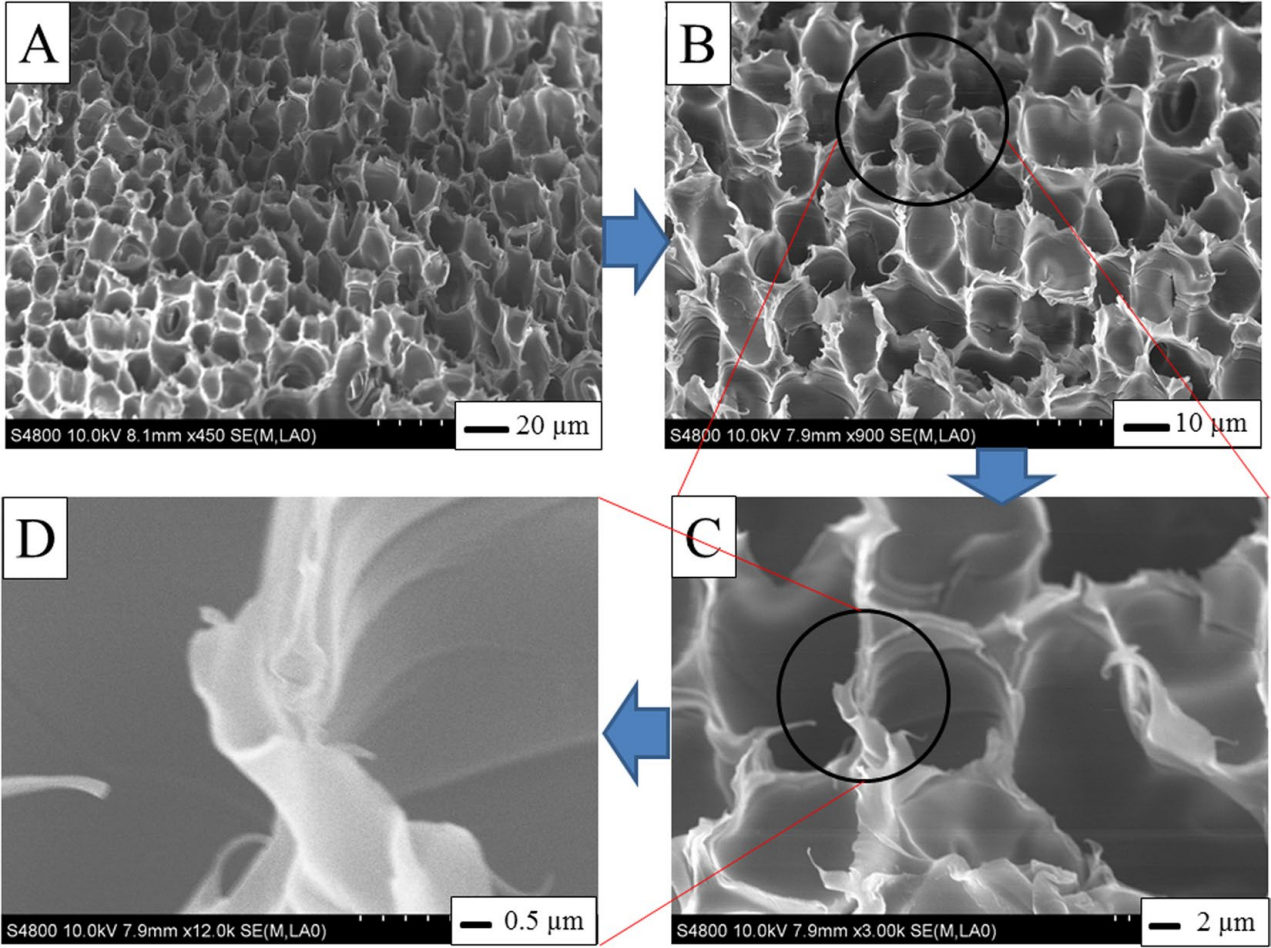
SEM images of a PS monolith at different magnifications.

To get faster removal, a porous structure with high surface area is quite important. The morphology of the PS monolith was observed by SEM. As shown in Fig. 2, the monolith had relatively uniform macrospores, with average pore sizes of 10 μm. The enlarged image (Fig. 2D) shows that the skeleton of the monolith was quite thin. The ultrathin skeleton and macroporous structure indicate that the monoliths had low density. The density was measured to be 0.096 g/cm^3^ and the porosity was determined as 92% by gravimetry using the equation described in the literature^33^. The macropore structure of the monolith is useful for the fast removal of oil. The mechanical property of the monolith is also important for the actual applications. The mechanical property of the obtained PS monolith is good enough for the absorption application (data not shown). The monolith is free-standing and can sustain the stirring force in the absorption process, indicating that the mechanical property of the monolith can fulfill the practical applications.

**Figure 3:**
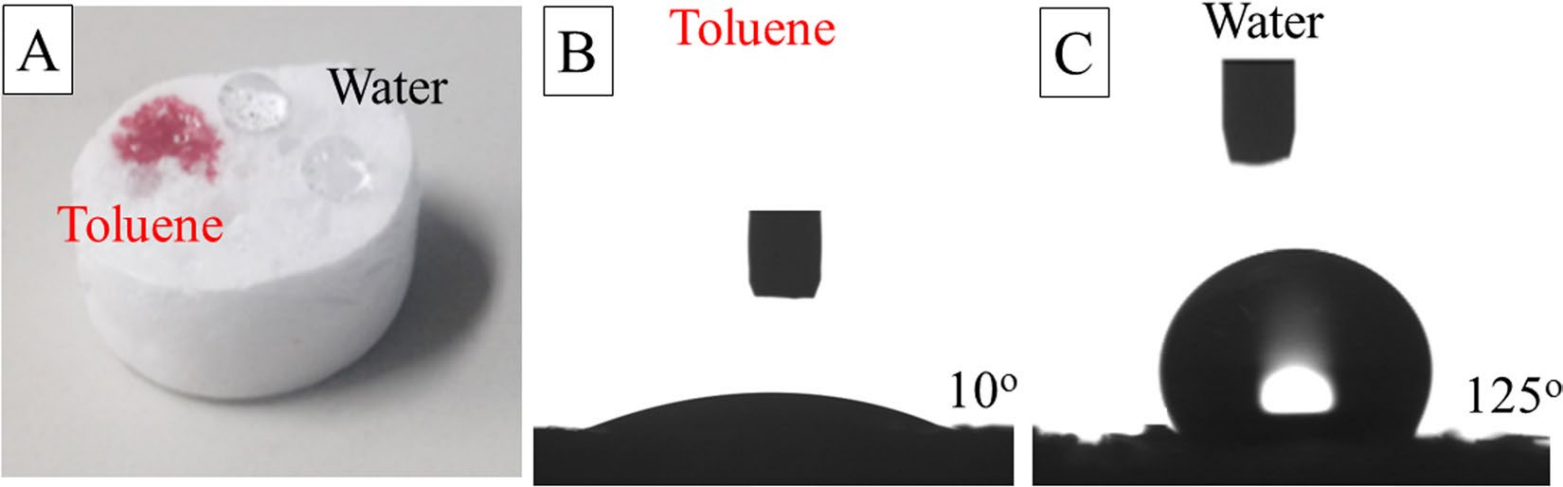
Droplets of toluene and water on the top of a PS monolith (**A**); the oil (**B**) and water (**C**) contact angles of the monolith.

The wetting property is also quite important for the oil absorption application. Figure 3B and C show the contact angles of the PS monolith toward oil and water, respectively. The water contact angle was measured as 120°, indicating the high hydrophobicity of the monolith. The high hydrophobicity demonstrates that the monolith can selectively repel water phase. At the same time, the oil contact angle was determined as 10°, indicating that the present monolith has the absorption ability toward oil.

### Separation of oil-in-water emulsion by PS monolith

**Figure 4:**
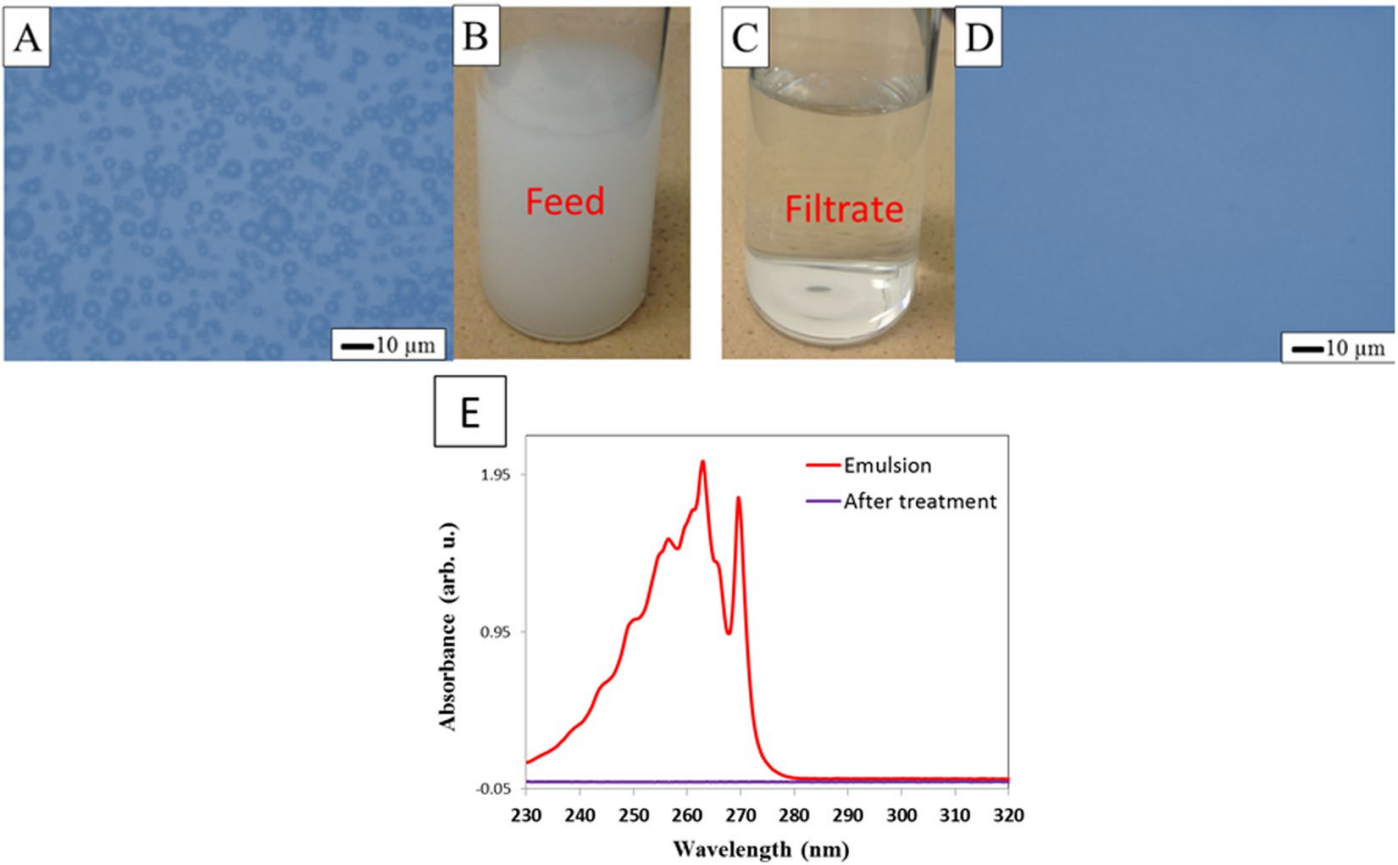
The photos of toluene-in-water emulsions before (**B**) and after separation (**C**), and the optical microscopy images of toluene-in-water emulsions before (**A**) and after separation (**D**), the UV absorption spectra of the emulsions before and after adsorption (**E**).

The specific wetting property of the PS monolith provides outstanding basis for the removal of oil-in-water emulsion. Figure 4 shows the separation result of toluene-in-water emulsions. The milky white color of the feed solution indicates the existence of colloidal toluene (Fig. 4B). The optical microscopy image illustrates that the sizes of colloids are around several micrometers (Fig. 4A). After absorption, the solution became transparent, similar to the color of pure water (Fig. 4C). In addition, no droplet was observed in the optical microscopy image (Fig. 4D), strongly implying that the toluene in the emulsion has been successfully removed. The UV absorption spectra were measured to check the removal of colloidal toluene. As shown in Fig. 4E, before absorption, the characteristic absorption peaks of toluene were observed. However, none of the peaks was observed in the spectrum of the removed aqueous solution, strongly indicating the high efficiency of the removal process. GC was applied to accurately measure the residual concentration of toluene. The toluene content in the filtrate was as low as 40 ppm. The solubility of toluene in water at room temperature is 50 ppm. Those results strongly demonstrate that not only the colloidal toluene but also part of dissolved toluene was removed by the PS monolith, indicating the high absorption efficiency. The high efficiency of the PS monolith depends on the strong interaction of the polymer toward the oils.

**Figure 5:**
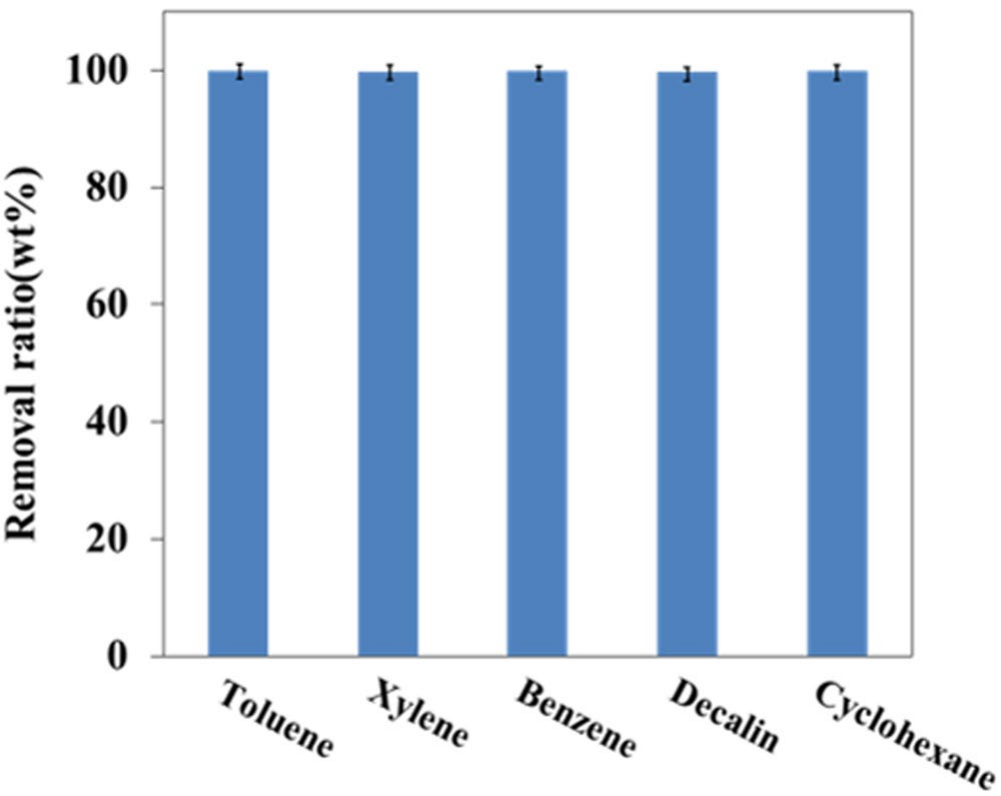
Absorption efficiency of the PS monolith toward emulsions prepared from different oils.

To determine the general absorption ability of the PS monolith, several common organic liquids were selected to prepare their corresponding colloidal emulsions. For all the above organic liquids, colloidal emulsions were formed with the droplets of several micrometers. Figure 5 shows the removal efficiency of the PS monolith toward different emulsions. In all the cases, The PS monolith exhibited high removal efficiency (more than 99%), strongly indicating that the absorption is general and the monolith is useful for the treatment of practical spilled oil which always contains several types of oils.

**Figure 6:**
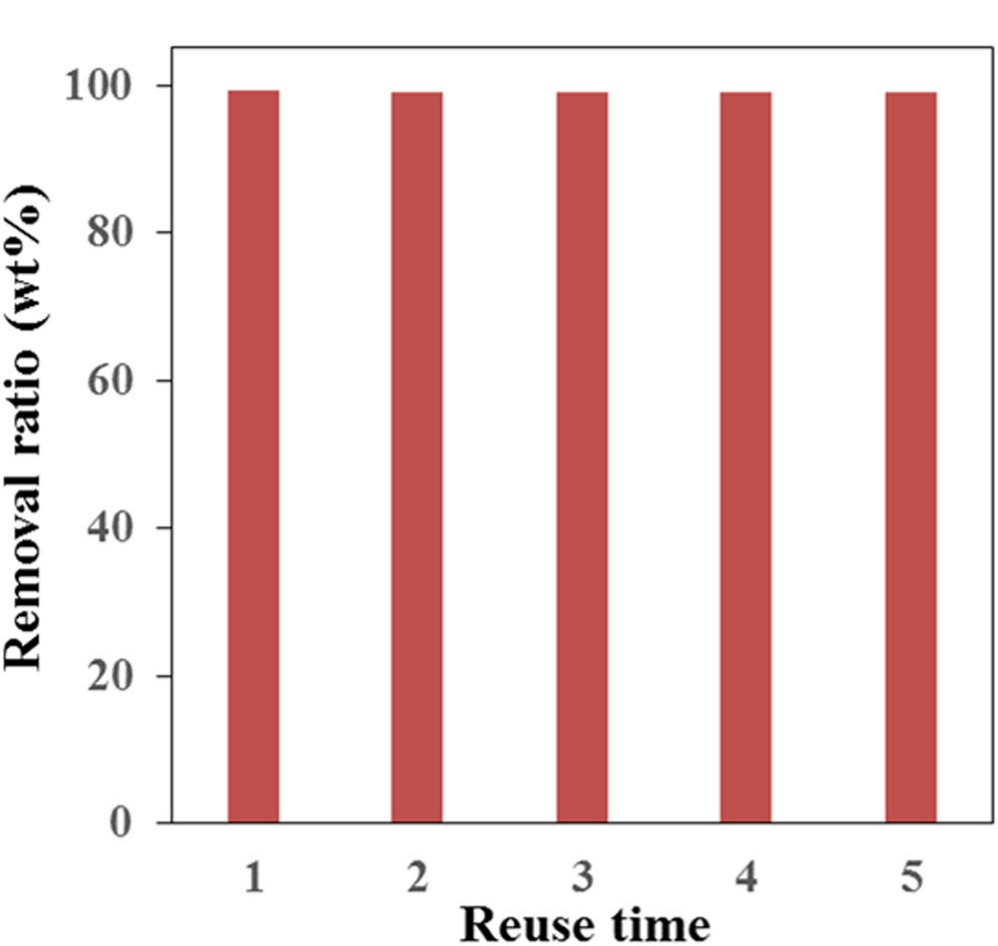
The removal ratio of toluene by the reused PS monolith for 5 times after vacuum drying between each cycle.

To demonstrate the possibility to reuse the monolith, the monolith was immersed in plenty of 2-propanol. Subsequently, the monolith was dried under vacuum for 4 hours. After drying, the monolith was used for another cycle of absorption, we found that the monolith can be used for another time, and can recover the oil in high efficiency. In a similar way, the monolith can remove the colloid toluene for more than 5 times, still with high efficiency (Fig. 6). This means the combination of solvent exchange and vacuum drying is an effective method to remove the absorbed oils and release the polystyrene for another absorption cycle. The polystyrene is a polymer that has strong interaction with the oils, making the monolith high affinity to adsorb them, leaving the residual oil of a quite low concentration, which is significant for the high-efficient treatment of oil spill accident. However, the monolith cannot be used to absorb a large amount of oils at one time, in which case, the structure of the monolith will be destroyed. This issue should be dissolved by further crosslinking of the monolith using some crosslinking method, such as the electron beam crosslinking. The further study is under development in our group.

## Conclusions

We have developed a facile and template-free method to synthesize a PS monolith with macroporous structure from its polymer solution. The fascinating properties of the fabricated monolith including macroporous structure, high hydrophobicity, and superoleophilicity, are suitable for colloidal oil treatment. Because of its strong interaction with the oils, the monolith can efficiently remove colloidal oils. Considering that the monolith is fabricated from polymer (PS) but not from monomers (styrene), the monolith should contribute to the recycle of waste PS plastic. We believe that the present PS monolith will provide us a new tool for oil spill treatment and will contribute to the industrial application.

## Materials and Methods

### Reagents

Decalin was supplied from Kanto Chemicals Co Inc. Polystyrene (Degree of polymerization: 2000), acetone, oil red, and 1-butanol were purchased from Wako Co. All reagents were used as received without further purification.

### Fabrication of PS monolith

We prepared the PS monolith by the following procedure (Fig. 1). Three hundred milligram of PS was dissolved in a mixed solvent containing 1 mL of decalin and 1 mL of 1-butanol at 115 °C. Subsequently, the solution was put at 20 °C to induce phase separation. The obtained monolith was immersed into 2-propanol to remove the embedded solvent. The monolith was dried under vacuum for further characterization and application.

### Emulsion absorption experiment

The oil-in-water emulsions were prepared by adding a certain amount of organic solvents (1 wt%) to water. Subsequently, the mixtures were emulsified for 5 minutes by a tip sonicator (580 W, 30% of amplification). The obtained emulsion is stable for at least 1 hour without any significant phase separation. To carry the absorption measurement, 0.5 g of PS monolith was cut into several pieces and was placed in a glass beaker filled with 5 ml of emulsions. The solution was stirred at a constant low speed. After absorption, the residual oil concentration in the water was determined using gas chromatography.

To demonstrate the recovery of the monolith, the monolith together with the absorbed oils was immersed in plenty of 2-propanol for solvent exchange. Subsequently, the monolith was put into the oven. After vacuum drying for 4 hours, the monolith was used for another cycle of adsorption.

### Characterizations

The scanning electron microscope (SEM, Hitachi S-4800) was used to observe the morphology of the PS monolith at an accelerating voltage of 10 kV. The monoliths were fractured to small pieces with the assistance of liquid nitrogen and fixed on a SEM sample stage. Subsequently, a thin layer of Pt film was sputtered onto the upper surface of the monoliths. Contact angles were measured using an OCA20 contact-angle system (Data-physics, Germany). The concentration of residual oils in the aqueous phase was measured using gas chromatography (Shimadzu, GC-2014). Optical microscopy images of the emulsions were taken using a BX 51TF Instec H601 (Olympus, Japan).
